# The impact of work-family conflict on the mental health of female employees in the financial sector of China: a cross-sectional study

**DOI:** 10.3389/fpubh.2026.1807662

**Published:** 2026-05-20

**Authors:** Yan Chen, Jie Tian, Kaixuan Huang, Ni Song, Zhe Zhou, Zhongxia Shen

**Affiliations:** 1 Zhejiang Provincial Committee of the China Finance and Trade Union, Hangzhou, China; 2 Zhejiang Communications Investment Group Co., Ltd., Hangzhou, China; 3 Yangtze Delta Region Institute of Tsinghua University, Jiaxing, China; 4 School of Engineering, Huzhou Normal University, Huzhou, China; 5 Department of Neurosis and Psychosomatic Diseases, Huzhou Third Municipal Hospital, The Affiliated Hospital of Huzhou University, Huzhou, China

**Keywords:** female employees, financial sector, mental health, occupational health, work-family conflict

## Abstract

Mental health among female financial employees is a critical occupational concern, yet the role of work-family conflict in this high-pressure field remains underexplored. This study assesses the mental health status of 2,342 female employees in China's financial sector and examines the specific impact of work-family conflict. Participants completed the Patient Health Questionnaire, Generalized Anxiety Disorder Scale, Insomnia Severity Index, and Perceived Stress Scale, while work-family conflict was measured with the Work-Family Conflict Scale. Results revealed elevated prevalence rates: depression (74.64%), anxiety (66.78%), insomnia (53.33%), and perceived stress (73.40%). Multivariate regression identified work-family conflict as a significant predictor of poorer mental health. This association persisted after controlling for demographic and occupational variables. The findings highlight work-family conflict as a key occupational risk factor. Implementing structured organizational measures, such as flexible work arrangements, is recommended to safeguard the wellbeing of this workforce.

## 1 Introduction

Mental health constitutes a critical global public health concern, with women's mental health issues being particularly prominent. Data from the China Mental Health Survey (CMHS) indicate that approximately 65% of people with depression in China are women, and the lifetime prevalence of depressive disorders among women is 1.44 times that of men (1). As essential contributors to both families and society, women's mental health status affects not only individual quality of life, but also family functioning and child development. A review of the existing literature reveals that women's mental health is shaped by multiple factors, including individual characteristics, family dynamics, and environmental conditions.

From an individual perspective, Hill and Angel (2) indicate that women generally report poorer mental health than men, with remarried women facing a particularly heavy psychological burden. Beyond individual factors, workplace stressors rooted in traditional gender roles significantly increase women's risk of depression and anxiety (3). These findings demonstrate that gender, age, marital status, and workplace stress exert substantial influence on women's mental health.

From a family perspective, modern women frequently navigate multiple roles, balancing paid employment with unpaid domestic labor. A study of German mothers revealed that women fulfilling both “working” and “mothering” roles experience significant role strain. Non-specific maternal pressures, lack of autonomy, overprotective behaviors, and dissatisfaction with work-life balance all adversely impact their mental health (4). Longitudinal data indicate that women's dissatisfaction with the division of household labor and perceptions of inequity are associated with poorer mental health, with the burden of unpaid labor being a major contributor to psychological issues (5). A study of female university lecturers in China further confirmed that work-family conflict mediates the relationship between workplace gender barriers and occupational burnout, underscoring its central role in negative psychological outcomes among working women (6).

From an environmental perspective, studies based on national survey data in China show that social networks and mental health are key determinants of subjective wellbeing, with social networks mediating the relationship between health literacy and subjective wellbeing (7). The erosion of social networks significantly undermines mental health (8). Research on female casino employees revealed that collegial support substantially alleviates work-family conflict, suggesting workplace social support plays a vital role in mitigating work-family pressures among professional women (9). Analysis of the Korean National Mental Health Survey demonstrated that social isolation (OR = 4.44) is significantly correlated with suicidal behavior, indicating its pronounced negative impact on mental health (10). Moreover, environmental stressors have been shown to be strongly associated with heightened depressive symptoms and increased suicidal ideation among specific female cohorts (11).

Recent empirical studies have confirmed the significant impact of work-family conflict on the mental health of Chinese career women across various industries. However, the financial sector presents a unique and highly demanding occupational context. Characterized by intense market competition, rigid performance metrics, and a culture of prolonged working hours, the financial industry exposes employees to severe occupational stress. Such environment is strongly correlated with elevated risks of depression, anxiety, and insomnia (12). For female financial professionals, the collision between these high-stakes professional demands and traditional domestic responsibilities creates a severe work-family conflict, often leading to accelerated psychological ego-depletion. Despite these documented risks, a critical research gap remains. Most existing occupational health studies tend to treat the financial sector as a homogeneous entity or focus primarily on general stress, lacking specific exploration of female-centric psychosocial hazards. Furthermore, there is a paucity of empirical evidence regarding how different sub-sectors within finance and complex family dynamics differentially impact women's mental health in the Chinese context. Therefore, this study investigates the prevalence of mental health problems among female financial employees in China. The novelty of this research lies in: (1) quantifying the relative weight of work-family conflict against other demographic factors within this specific high-pressure environment; and (2) uncovering the nuanced, sub-sector-specific mechanisms that drive psychological distress. By doing so, this study provides targeted, empirical evidence essential for developing tailored occupational health interventions and policies for women in the financial sector.

## 2 Methods

### 2.1 Study design

This study employs a quantitative cross-sectional survey design initiated in July 2025. The research design phase (July–August 2025) encompassed a systematic literature review, field research, and the selection and validation of measurement tools. Following rigorous psychometric evaluation, the Patient Health Questionnaire (PHQ-9), Generalized Anxiety Disorder Scale (GAD-7), Insomnia Severity Index (ISI), Perceived Stress Scale (PSS-10), and Work-Family Conflict Scale (WAFCS) were finalized as core assessment tools.

### 2.2 Sample size calculation

The sample size calculation is based on the statistical requirements of multiple linear regression analysis. The regression model in this study includes nine predictor variables: work-family conflict scores (one continuous variable), number of children (four categories, coded as three dummy variables), and sector type (six categories, coded as five dummy variables). According to Green's sample size calculation guidelines for multiple regression (13), the minimum sample size required to test overall model goodness-of-fit is *N*≥50+8*k* (where *k* is the number of predictor variables), i.e., *N*≥122. The minimum sample size required to test the significance of individual regression coefficients is *N*≥104+*k*, i.e., *N*≥113. Furthermore, to ensure sufficient statistical power (1−β≥0.80) for detecting a moderate effect size (*f*^2^ = 0.15) at a significance level of 0.05, a prior power analysis indicated a required sample size of approximately 103 cases. Considering the aforementioned statistical requirements, potential data loss (estimated at 5%–10%), and the need for stratified analysis across different subgroups, this study sets a target sample size of no fewer than 800 subjects.

### 2.3 Participants and data collection

Given the strict data confidentiality and privacy regulations within the financial sector, obtaining a complete randomized roster of all employees was unfeasible. Therefore, convenience sampling via the coordination of trade unions was deemed the most practical and effective approach to access this specific occupational population. Using convenience sampling, an online survey was administered to female employees in the financial sector of Zhejiang Province, China, through organizational coordination between September 14 and 24, 2025. A pilot test (*n* = 50) was conducted prior to distribution to assess feasibility and comprehensibility. The formal survey distributed 2,440 questionnaires, with 2,342 valid responses collected, yielding a response rate of 95.98%. Data was excluded based on the following criteria: abnormally short (<3 min) or long (>60 min) response times, consecutive identical selections across multiple items, or missing values exceeding 10% for key variables.

The final sample size (*N* = 2, 342) was 21.1 times the theoretical minimum, significantly exceeding the preset target. To mitigate potential sampling bias inherent in convenience sampling, we strictly controlled the inclusion criteria and maximized the sample size across multiple financial sub-sectors. A *post-hoc* power analysis confirmed that with the current sample, the study possesses power near 1.0 to detect small-to-medium effect sizes (*f*^2^≥0.02), ensuring precision and stable parameter estimation, sufficient power to detect effects of key predictors, and robust findings with good external validity.

The research subjects primarily comprised ordinary frontline employees (84.33%), characterized by being predominantly middle-aged and young adults (31–40 years old, 37.53%), highly educated (bachelor's degree or higher, 90.65%), working within the banking system (70.79%), married (70.45%), and having a small number of children (one child, 47.48%).

### 2.4 Measures

A self-reported questionnaire collected information on age, education, marital status, number of children, sector affiliation, and job position.

Mental health and work-family conflict were evaluated using five validated self-report scales. The 10-item Perceived Stress Scale (PSS-10; α = 0.849) assessed stress levels, with a total score >13 indicating perceived stress (14). Anxiety was screened using the 7-item Generalized Anxiety Disorder scale (GAD-7; α = 0.950), where scores >4 denote anxiety (15). Depression risk was evaluated by the 9-item Patient Health Questionnaire (PHQ-9; α = 0.917), using a cut-off of >4 (16). Insomnia was measured via the 7-item Insomnia Severity Index (ISI; α = 0.890), with scores >7 indicating clinically significant insomnia (17). Work-family conflict was assessed using the 10-item Work-Family Conflict Scale (WAFCS; α = 0.898), where higher scores (range 10–50) reflect greater conflict (18). A composite mental health score was computed by averaging the standardized scores of the four outcome measures: depression, anxiety, insomnia, and perceived stress. Work-family conflict was analyzed as a separate, independent predictor. The composite score showed excellent reliability (Cronbach's α = 0.9469).

### 2.5 Statistical analysis

Data were analyzed using Python, version 3.9 (Python Software Foundation, Beaverton, OR, USA). Categorical data are presented as frequencies and percentages. Continuous data meeting normality assumptions are described as mean ± standard deviation. Group comparisons were performed using one-way analysis of variance (ANOVA). Multivariate linear regression was used to examine influencing factors. A *p* value <0.05 was considered statistically significant.

## 3 Results

### 3.1 Mental health status of female employees in the financial sector

Among the female financial sector employees, 1,748 individuals (74.64%) were identified as at risk for depression, with a mean score of 8.09 ± 5.41. Symptoms of anxiety were present in 1,564 individuals (66.78%), with a mean score of 6.45 ± 4.76. Clinical insomnia was detected in 1,248 participants (53.33%), mean score 8.30 ± 4.89. Perceived stress was reported by 1,719 individuals (73.40%), mean score 17.08 ± 6.81.

The sample primarily consisted of frontline employees (84.33%), who were predominantly middle-aged (31–40 years old, 37.53%), highly educated (bachelor's degree or above, 90.65%), employed in the banking sector (70.79%), married (70.45%), and had one child (47.48%).

### 3.2 Univariate analysis of mental health status

One-way ANOVA was conducted to examine differences in composite mental health scores across demographic variables (age, education, marital status, number of children, sector, and position). The results (Table 1) indicated statistically significant differences in mental health based on the number of children (*F* = 3.67, *p* = 0.012) and financial sub-sector (*F* = 2.39, *p* = 0.036). No significant differences were found for age, education, marital status, or position (*p*>0.05).

**Table 1:** Single-factor analysis of mental health status.

Group	Options	Number of people (Percentage)	Mental health score (*M*±*SD*)	*F* value	*p* Value
Age	25–30 years old	667 (28.48%)	33.63 ± 16.53	2.07	0.103
	31–40 years old	879 (37.53%)	33.71 ± 15.67		
	41–50 years old	619 (26.43%)	32.99 ± 16.26		
	50 years old and above	177 (7.56%)	30.57 ± 15.27		
Education	Associate degree or below	219 (9.35%)	30.97 ± 16.80	2.75	0.064
	Undergraduate degree	1,869 (79.80%)	33.60 ± 15.87		
	Graduate student	254 (10.85%)	32.77 ± 16.64		
Marital status	Married	1,650 (70.45%)	33.00 ± 16.06	1.47	0.230
	Unmarried	592 (25.28%)	33.57 ± 16.08		
	Divorced	100 (4.27%)	35.69 ± 15.85		
Number of children	0	729 (31.13%)	33.85 ± 15.98	3.67	0.012
	1	1,112 (47.48%)	33.56 ± 16.26		
	2	486 (20.75%)	32.04 ± 15.66		
	Three or more	15 (0.64%)	22.45 ± 12.61		
Financial sector	Bank	1,658 (70.79%)	33.85 ± 16.18	2.39	0.036
	Insurance	436 (18.62%)	31.25 ± 16.47		
	Securities	123 (5.25%)	34.57 ± 15.37		
	Fund	17 (0.73%)	30.74 ± 15.30		
	Futures	17 (0.73%)	30.80 ± 13.63		
	Others	91 (3.88%)	31.37 ± 12.14		
Position	Regular employee	1,975 (84.33%)	33.10 ± 16.10	0.71	0.493
	Department Head	355 (15.16%)	34.17 ± 15.92		
	Leadership	12 (0.51%)	31.92 ± 13.72		

### 3.3 Multivariate analysis of mental health

Multiple linear regression was performed with the composite mental health score as the dependent variable, work-family conflict score as the independent variable, and the number of children and sector as covariates (Table 2).

**Table 2:** Multivariate linear regression of mental health.

Independent variables and constants	Options	β value	Standard error	*t* Value	*p* Value	95%CI
Constants		0.015	0.034	0.426	0.670	−0.053 to 0.082
Number of children^a^	1	0.030	0.042	0.708	0.479	−0.053 to 0.113
	2	−0.046	0.052	−0.883	0.377	−0.147 to 0.056
	Three or more	−0.671	0.230	−2.916	0.004	−1.122 to −0.220
Financial sector^a^	Fund	−0.284	0.215	−1.321	0.187	−0.705 to 0.138
	Futures	0.031	0.215	0.144	0.886	−0.391 to 0.453
	Insurance	−0.109	0.048	−2.285	0.022	−0.202 to −0.015
	Securities	0.188	0.083	2.274	0.023	0.026–0.350
	Others	−0.071	0.095	−0.747	0.455	−0.257 to 0.115
Work-family conflict		0.469	0.018	25.598	<0.001	0.433–0.505

^a^The number of children is set to zero, and the bank is used as the reference.

Work-family conflict was a significant predictor of mental health (β = 0.469, *p* < 0.001), indicating that higher conflict levels were associated with poorer mental health.

Regarding the number of children, having three or more children was associated with significantly better mental health compared to having no children (β = −0.671, *p* = 0.004).

For sector type, working in the insurance sector was associated with better mental health relative to banking (β = −0.109, *p* = 0.022), whereas working in the securities sector was associated with poorer mental health (β = 0.188, *p* = 0.023).

## 4 Discussion

This study systematically reveals the severe mental health status and its influencing factors among female employees in the financial sector through a survey of 2,342 women in Zhejiang Province, China. Findings indicate that the prevalence rates of depression, anxiety, insomnia, and perceived stress among female financial workers reached 74.64%, 66.78%, 53.33%, and 73.40%, respectively. This suggests that female financial workers have become a high-risk group for mental health issues, similar to findings from studies on other high-stress occupational groups such as healthcare workers (19), demanding urgent attention from society and organizations. Both univariate and multivariate analyses indicate that number of children, sector type, and work-family conflict are significant factors affecting the mental health of female employees in the financial sector, with work-family conflict being the most pronounced.These findings align with research demonstrating the complex pathways through which family-related factors influence mental health outcomes (20). This section will first discuss the differential effects of number of children and sector type, then focus on analyzing the core mechanisms and health risks associated with work-family conflict, and finally propose targeted intervention recommendations.

### 4.1 Core mechanisms of work-family conflict and associated health risks

After controlling for demographic variables, work-family conflict (WFC) emerged as the predictor of mental health issues (β = 0.469, *p* < 0.001). Drawing upon the foundational Conservation of Resources theory (21), and supported by recent empirical evidence on occupational burnout in China (22), the detrimental impact of WFC on female financial workers can be elucidated through three pathways: (1) the zero-sum game of time resources: the financial industry's pervasive culture of overtime and frequent travel structurally encroaches on family time, creating chronic role strain; (2) the continuous depletion of psychological energy: the rapid transition between high-stakes professional excellence and demanding family caregiving imposes a dual cognitive and emotional load, accelerating psychological burnout; and (3) internalized expectation conflict: the clash between a highly competitive workplace culture and traditional gender role expectations often places women in a “lose-lose" dilemma, leading to profound guilt and anxiety (23).

Sustained WFC not only directly triggers emotional disorders but also acts as a catalyst for clinical insomnia, which further impairs emotional regulation. For financial institutions, ignoring these pervasive mental health risks inevitably translates to decreased productivity, elevated turnover rates, and increased corporate healthcare costs.

### 4.2 Differential effects of children and sector type on mental health

Our analysis revealed counterintuitive findings regarding family size: female employees with three or more children exhibited significantly better mental health than those without children. While contradicting the conventional more children equal more stress paradigm, this aligns with recent perspectives on maternal resilience and sibling support dynamics. Larger families may foster robust internal support networks where older siblings share caregiving burdens (24). Furthermore, mothers with multiple children have typically navigated critical career-building phases and developed more mature emotional regulation and time-management strategies. However, due to the small sample size of this specific subgroup, these findings should be interpreted with caution and require further validation.

Regarding industry type, significant sub-sector heterogeneity was observed. Female employees in the securities sector reported the highest psychological pressure, whereas those in the insurance sector reported the lowest. This disparity can be structurally explained through Karasek's classic Job Demand-Control model (25). The securities sector is characterized by extreme market volatility, short-term performance evaluations, and high uncertainty (high demand, low control), which inherently exacerbates psychological distress. Conversely, the insurance sector often affords greater flexibility in work hours and geographic mobility (higher control), thereby buffering the negative impacts of occupational stress.

### 4.3 Practical insights and intervention recommendations

To mitigate these critical occupational hazards, generalized support is insufficient. We propose the following evidence-based interventions: first, establish routine, confidential mental health screening mechanisms within financial institutions to identify high-risk individuals early. Second, implement proven, structured family-friendly policies. Recent comprehensive reviews demonstrate that formalized Flexible Work Arrangements, such as telecommuting and flexible scheduling, significantly reduce WFC by enhancing employees' perceived autonomy and wellbeing (26). Implementing “family leave hour banks" and destigmatizing their use can provide crucial buffers during family emergencies. Third, tailor interventions to specific sub-sectors: for instance, focusing on performance-evaluation reform in the securities sector, while enhancing boundary-management training in the insurance sector.

### 4.4 Research limitations and future prospects

Several limitations must be acknowledged. First, due to the cross-sectional design of this study, we can only identify correlational associations between work-family conflict, demographic factors, and mental health outcomes; strict causal relationships cannot be inferred. Longitudinal studies are required to confirm causality. Second, all data were collected via self-report questionnaires, which may introduce common method bias and social desirability bias, potentially overestimating or underestimating symptom severity. Finally, the use of convenience sampling within a single province (Zhejiang) may limit the generalizability of the findings to female financial employees in other regions or countries with different socio-economic dynamics. Future research could utilize randomized, multi-center sampling to validate these findings. Further, longitudinal designs with multiple time points could be employed to track the dynamic interaction between work-family conflict and mental health, helping to reveal how such psychological issues emerge, evolve, and potentially resolve over time.

## Data Availability

The original contributions presented in the study are included in the article/supplementary material, further inquiries can be directed to the corresponding author.
